# Sex-Related Differential Whole-Brain Input Atlas of Locus Coeruleus Noradrenaline Neurons

**DOI:** 10.3389/fncir.2020.00053

**Published:** 2020-09-23

**Authors:** Pei Sun, Junjun Wang, Meng Zhang, Xinxin Duan, Yunfei Wei, Fuqiang Xu, Yan Ma, Yu-Hui Zhang

**Affiliations:** ^1^Britton Chance Center for Biomedical Photonics, Wuhan National Laboratory for Optoelectronics – Huazhong University of Science and Technology (HUST), Wuhan, China; ^2^MoE Key Laboratory for Biomedical Photonics, School of Engineering Sciences, Huazhong University of Science and Technology, Wuhan, China; ^3^Centre for Brain Science, State Key Laboratory of Magnetic Resonance and Atomic Molecular Physics, Key Laboratory of Magnetic Resonance in Biological Systems, Wuhan Institute of Physics and Mathematics, CAS Centre for Excellence in Brain Science and Intelligence Technology, Chinese Academy of Sciences, Wuhan, China; ^4^HUST-WHBC United Hematology Optical Imaging Center, Wuhan Blood Center (WHBC), Wuhan, China

**Keywords:** sex differences, locus coeruleus noradrenaline neurons, whole-brain, monosynaptic tracing, quantitative analysis

## Abstract

As the most important organ in our bodies, the brain plays a critical role in deciding sex-related differential features; however, the underlying neural circuitry basis remains unclear. Here, we used a cell-type-specific rabies virus-mediated monosynaptic tracing system to generate a sex differences-related whole-brain input atlas of locus coeruleus noradrenaline (LC-NE) neurons. We developed custom pipelines for brain-wide comparisons of input sources in both sexes with the registration of the whole-brain data set to the Allen Mouse Brain Reference Atlas. Among 257 distinct anatomical regions, we demonstrated the differential proportions of inputs to LC-NE neurons in male and female mice at different levels. Locus coeruleus noradrenaline neurons of two sexes showed general similarity in the input patterns, but with differentiated input proportions quantitatively from major brain regions and diverse sub-regions. For instance, inputs to male LC-NE neurons were found mainly in the cerebrum, interbrain, and cerebellum, whereas inputs to female LC-NE neurons were found in the midbrain and hindbrain. We further found that specific subsets of nuclei nested within sub-regions contributed to overall sex-related differences in the input circuitry. Furthermore, among the totaled 123 anatomical regions with proportion of inputs >0.1%, we also identified 11 sub-regions with significant statistical differences of total inputs between male and female mice, and seven of them also showed such differences in ipsilateral hemispheres. Our study not only provides a structural basis to facilitate our understanding of sex differences at a circuitry level but also provides clues for future sexually differentiated functional studies related to LC-NE neurons.

## Introduction

Sex differences, i.e., differences between male and female organisms, are prevalent among a number of organs, including the liver, kidney, muscles, gut, and brain (De Vries and Forger, 2015). Recently, much more attention has been placed on introducing sex as a biological variable in basic and preclinical animal research (Cahill, 2006; Shansky and Woolley, 2016; Joel and McCarthy, 2017). In the mammal brain, an exponentially increasing body of evidence indicated that striking sex differences exist among multiple behaviors, such as cognitive function, fear, stress, and pain (Andreano and Cahill, 2009; Farrell et al., 2013; Bangasser et al., 2016; Sorge and Strath, 2018), as well as several neurological and psychiatric diseases, such as Parkinson’s disease, Alzheimer’s disease, and epilepsy (Zagni et al., 2016). For instance, females were more sensitive to pain and more prone to be victims of pain or pain-related syndromes (Nahman-Averbuch et al., 2015; Sorge and Strath, 2018). Several clinical studies demonstrate that compared with men, women were at 2–3 times higher risk of developing migraine, post-traumatic stress disorder, and Alzheimer’s disease (Olff, 2017; Vetvik and MacGregor, 2017; Laws et al., 2018); conversely, males were more prone to suffer from diseases such as Parkinson’s disease and autism spectrum disorder than females (Zagni et al., 2016). Numerous previous studies have shown that multiple brain regions, which are involved in these functions and in psychiatric disorders, such as the hypothalamus, bed nuclei of the stria terminalis (BST), hippocampal CA1, CA3, and dentate gyrus and locus coeruleus (LC), are sexually differentiated on both structural and functional levels (Hutton et al., 1998; De Vries and Simerly, 2002; Shah et al., 2004; Bangasser et al., 2016; Yagi and Galea, 2018).

Locus coeruleus, a brainstem nucleus, contains at least two different cell types, the thoroughly studied noradrenaline (LC-NE) neurons and the recently emphasized GABAergic neurons (LC-GABA). Locus coeruleus noradrenaline neurons belong to a family of neuromodulatory systems. Although they have a small population, they project to a variety of regions across the brain with ascending projections to the olfactory bulb, cerebral cortex, amygdala, hippocampus, and thalamus, and descending projections to the medulla and spinal cord. Reciprocally, LC-NE neurons receive a broad range of inputs from similar brain regions (Schwarz and Luo, 2015). Thus, it is unsurprising that LC-NE neurons play a critical role in diverse functions, including attention, anxiety, stress-response, arousal/sleep, learning and memory, sensory processing, pain modulation, and reward processing (Aston-Jones and Cohen, 2005; Sara, 2009; Carter et al., 2010; Hickey et al., 2014; Hofmeister and Sterpenich, 2015; Takeuchi et al., 2016; McCall et al., 2017; Li et al., 2018; Waterhouse and Navarra, 2019).

Due to the key role in regulating mammal neural networks, multiple studies have focused on LC and employed LC-NE neurons as paradigms for elucidating the underlying mechanisms of sex differences with regard to the effects of sex hormones, structural differences, and molecular mechanisms (Pinos et al., 2001; Garcia-Falgueras et al., 2005; Bangasser et al., 2011, 2016; De Carvalho et al., 2016; Mulvey et al., 2018). Among them, sex hormones have been shown to have a differential influence on the activities of LC-NE neurons in rats of different sexes (De Carvalho et al., 2016). Furthermore, previous structural studies generally focused on somal and dendritic differences of LC-NE neurons. For instance, female rats exhibit a larger LC size and more LC-NE neurons than male subjects, though these are defined to a limited strain or species (e.g., Wistar strain; Pinos et al., 2001; Garcia-Falgueras et al., 2005). The dendritic appearance of LC-NE neurons in female rats is morphologically more complex, with a higher density of dendrites, prolonged dendritic extensions leading to a larger coverage, and more dendritic branch points and ends for increased number of synaptic contacts (Bangasser et al., 2011). These structural differences, to some extent, can account for the different performances of some behaviors, such as arousal and stress-related psychiatric disorders between male and female subjects. Recently, a thorough transcriptional profiling survey with identification of more than 3,000 genes in LC have revealed substantial sex differences (>100 genes) of LC-NE neurons on a transcript level and further demonstrated that these differential gene expressions are able to generate sex-related different behavioral responses (Mulvey et al., 2018). However, to the best of our knowledge, no systematic and comprehensive analysis of brain-wide inputs to LC-NE neurons in male and female mice has been reported so far.

Herein, we used cell-type-specific rabies virus-mediated monosynaptic tracing systems to map the brain-wide input sources of LC-NE neurons in male and female mice. We registered the whole-brain to Allen Mouse Brain Reference Atlas, made quantitative comparisons of the input data sets at different levels, and generated the sex difference-related whole-brain atlas. We identified multiple presynaptic regions that showed substantial input differences in both ipsilateral and contralateral hemispheres. We further demonstrated that sex difference-related overall differential inputs to LC-NE neurons were derived from specific subsets of nuclei nested within respective sub-regions. Our study thus provided a structural basis for the further sex-involved functional studies targeting LC-NE neurons.

## Materials and Methods

### Mice and Viruses

We used 2-month-old male and female dopamine β-hydroxylase-Cre (Dbh-Cre) transgenic mice (Tg(Dbh-cre)KH212Gsat/Mmucd, Stock #032081-UCD, RRID: MMRRC_032081-UCD; Gong et al., 2007) in this study. Male and female littermates (*n* = 4 each) with similar weight (21–23 g) were chosen to make faithful comparisons. Adeno-associated virus (AAV) helpers used for rabies virus (RV)-mediated monosynaptic tracing were prepared according to our previous viral co-packaging strategy (our unpublished data)^1^. All vectors and viruses used in this study were provided by BrainVTA (BrainVTA Co., Ltd., Wuhan, China). Two vectors, AAV-EF1α-DIO-EGFP-TVA (GT, 1.0 μg/μl) and AAV-EF1α-DIO-RG (0.5 μg/μl), were premixed at a ratio of 1:2, and then the mixtures of the two plasmids were co-transfected with Ad helper vector and pAAV-rep/cap vector into HEK293T cells, followed by the standard processes of AAV production (Grieger et al., 2006). The resulted rAAV with serotype 2/9 was abbreviated for lAAV-DIO-GT/RG in convenience (l refers to littermate). The AAV titer was determined by quantitative real-time PCR and estimated to be 7.0 × 10^12^ viral genome (VG)/ml. Genetically modified RV (EnvA-SADΔG-DsRed) were prepared as previously described (Osakada and Callaway, 2013) and the titer was estimated to be 2.0 × 10^8^ infectious particles/ml. Animals were housed in a strict specified pathogen-free (SPF) environment with a 12 h:12 h light:dark cycle.

### Virus Injections, Perfusion, and Whole-Brain Sectioning

All animal experimental procedures were approved by the Institutional Animal Ethics Committee of Huazhong University of Science and Technology (HUST). The injection protocol was performed as follows. Mice were deeply anesthetized by 3% isoflurane in oxygen. A volume of 100 nl rAAV helpers and lAAV-DIO-GT/RG were injected into the right hemisphere of the LC (anterior/posterior (AP) – 5.4 mm, medial/lateral (ML) – 0.8 mm and dorsal/ventral (DV) – 3.7 mm) of the male and female mice unilaterally using stereotaxic apparatus (#68030, RWD life science, China). Three weeks later, 150 nl of genetically modified RV was injected into the same area of both groups. Viruses were all injected with a pulled glass micropipette at a rate of 10 nl/min for each injection and the micropipette was allowed to stay for an additional 5 min or more before withdrawal. After nine days’ infection of RV, all mice were transcardially perfused with 0.01 M phosphate buffered saline (PBS) followed by 4% paraformaldehyde (PFA) in 0.01 M PBS. The extracted brain samples were embedded with 4% agarose (V900510, Sigma-Aldrich, United States) in 0.01 M PBS and sectioned coronally at 50-μm thickness across the whole brain with VT1200S vibratome (Leica Biosystems, Germany). Coronal sections were sequentially collected to the 48-well plate with PBS. Every second section was chosen for analysis and counterstained with 4’,6’-diamidino-2-phenylindole (DAPI) to determine the cortical and laminar border, followed by mounting with covers glass using anti-fade fluorescence mounting medium (P0126, Beyotime Biotechnology, China) and sealing with nail polish for imaging.

### Whole-Brain Imaging, Registration, and Signal Identification

The whole-brain coronal sections were imaged with a VS120 slide scanner (Olympus, Japan) at 10× magnification. Three channels were used for imaging, with blue channel for DAPI staining, red channel for input cells, and the green channel for starter cells across the injection site. During the process of imaging, parameters were adjusted based on the input signals of specific sections. For sections with dense input labeling, we first imaged the whole coronal plane with a high exposure time to ensure cells with lower signals were captured and further reimaged the dense labeling areas with a much lower exposure time to make the input cells visually identifiable (for representative examples, see Supplementary Figure 1A).

The process of registration was based on several previously reported methods (Pollak Dorocic et al., 2014; Do et al., 2016; Fürth et al., 2018) and contained three steps: data set preparation, coarse matching, and fine adjustment. Data set preparation was performed using ImageJ software (National Institutes of Health, United States). Coarse matching and fine adjustment were performed using Photoshop CS6 software (Adobe Systems incorporated, San Jose, United States).

1. Data set preparation: a set of raw coronal images of a whole-brain [from the most anterior (+3.0 mm from the bregma) to the most posterior (−6.36 mm from the bregma)] were sequentially aligned from left to right. All sections were aligned in the same direction (the ipsilateral hemispheres were on the left) to distinguish the ipsilateral hemispheres (hemispheres with the injection site) and the contralateral hemispheres. Manual rotations were applied to some improperly placed sections. All the processed images were stored in JPEG format for subsequent matching. The online 2D version of the Allen Mouse Brain Reference Atlas (2011 Allen reference atlas)^2^ was used as the reference. The outlines of each plate were extracted from the Reference Atlas and stored in order as a separate file.
2. Coarse matching: the prerequisite of perfect registration was to apply the appropriate reference plates to the corresponding raw images. To facilitate the registration, we divided the whole brain into five parts based on the large landmarks: (a) Part I (+3.0 to +1.0 mm from the bregma): matching was based on the changing of the cerebral cortex (including prefrontal cortex and motor area) and the shape of the corpus callosum and anterior forceps (fa); (b) Part II (+1.0 to −1.0 mm from the bregma): matching was based on the changing of the ventral part of each coronal section and the shape of the lateral septal complex (LSX); (c) Part III (−1.0 to −3.8 mm from the bregma): matching was based on the shape of the hippocampus; (d) Part IV (−3.8 to −5.3 mm from the bregma): matching was based on the shape of the periaqueductal gray (PAG); (e) Part V (−5.3 to −6.36 mm from the bregma): matching was based on the shape of the fourth ventricle (V4). The brains were sliced at a 50-μm thickness and every other section was chosen for analysis, which was perfectly in accordance with the 100 μm interval between two adjacent Allen Mouse Brain Reference Atlas plates. Once the distance from the bregma of one section was defined, the residual sections can be defined accordingly. For each section, the size of the raw image was set to resemble the reference plate, making the following fine adjustment easier.
3. Fine adjustment: the coarse matching brain images went through free-form deformation to finely tone down diverse sub-regions by manipulating several grid points. We used the following information to assist the free-form deformation, including landmarks provided by DAPI staining and distribution of DsRed-labeled input signals and Allen online Nissl staining database^2^. Allen online Nissl staining database was particularly useful for midbrain regions (−4.0 to −5.0 mm from the bregma), where the landmarks defined by DAPI staining were not obvious and the DsRed-labeled input signals were extremely dense. Multiple easily identifiable landmarks were available for the fine adjustment, including the corpus callosum (cc, +1.0 to −4.5 mm from the bregma), interal capsule (int, 0 to −1.9 mm from the bregma), and cerebal peduncle (cpd, −1.9 to −2.9 mm from the bregma). For instance, cc is a representative landmark that distinguishes the cortical regions from the subcortical regions; int was used to distinguish the caudoputamen (CP) from the thalamus and cpd was used to distinguish the amygdala from the hypothalamus.

Manual identification of the input cells was performed using a multi-point tool in the ImageJ software (National Institutes of Health, United States). Identification of the positive signals, i.e., DsRed-labeled input cells with apparent cell bodies, were counted by the smart judgment of two experienced annotators who were blind to the sex of the mice. Several criteria were used to distinguish the positive signals from artificial signals, including the shape and size of cell bodies, whether with apparent dendrites protruding from cell bodies or not. For example, cell bodies were typically of irregular shapes whereas noise signals were more frequently found with a round shape; cell bodies typically had more than one protruding dendrite whereas noise signals did not. Moreover, the prevalent, brightly labeled dendrites whose cell bodies were cut off due to the mechanical sectioning caused great confusion to cell counts, which, however, could be solved by the size of the structure since cell bodies typically had a larger diameter than dendrites (Supplementary Figure 1B). Input cells located within space areas of two adjacent brain regions were regarded as a part of proximal regions according to previous reports (i and ii in Supplementary Figure 1C) (Schwarz et al., 2015). Input cells located within blank areas, i.e., brain regions with no definitions in the Allen Mouse Brain Reference Atlas, were counted separately and were not assigned to any brain regions (iii in Supplementary Figure 1C). To avoid background labeling, similar areas with a coverage of injection site (LC) and LC-adjacent brain regions (−4.96 to −5.78 mm from the bregma), including minority parts of the parabrachial nucleus (PB), midbrain reticular nucleus (MRN), supratrigeminal nucleus (SUT), and pontine reticular nucleus (PRNr), were excluded from the final analysis for both groups (Supplementary Figure 1D). With the exception of these regions, DsRed-labeled input cells, denoted by a yellow cross symbol in the centre of cell bodies in ImageJ, were counted throughout the entire brain.

Quantification of the starter cells was based on the co-expression of EGFP and DsRed. In both male and female groups, the vast majority of the starter cells were located in the LC (Supplementary Figure 2A). A very minority of starter cells were observed in A7 (male group: 5.61 ± 0.82% and female group: 6.42 ± 1.19%; Supplementary Figure 2B). Because the populations of the starter cells in A7 were small and similar in both male and female groups, we excluded these neurons from the final analysis. No starter cells were observed in A5 (Supplementary Figure 2C).

### Data Analysis

For the generation of sex differences-related whole-brain input atlas of LC-NE neurons, brain-wide quantitative analysis regarding the proportion of inputs in each anatomical region (that is, the number of input neurons in that region over the total number of input neurons) were performed for both male and female groups. Anatomical definitions and anatomical classifications of diverse brain regions were all in accordance with the Allen Mouse Brain Reference Atlas. All quantitative data were presented as the mean ± s.e.m. Significance was analyzed using Mann–Whitney U test in GraphPad Prism version 6.0 (GraphPad Software Inc., San Diego, CA, United States).

## Results

### Strategies for Tracing Inputs to Sex-Related Differences in LC-NE Neurons

**FIGURE 1:**
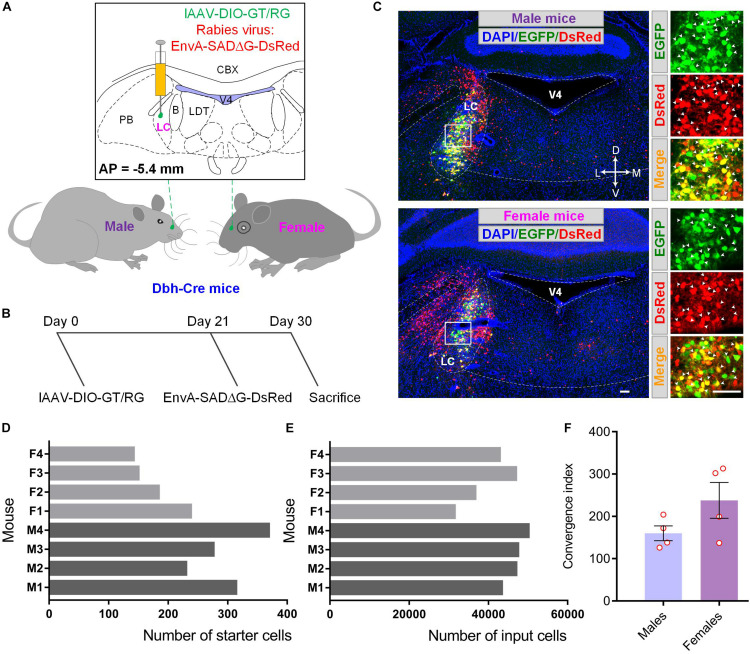
Demonstration of monosynaptic tracing systems to reveal the sex-related differences in LC. **(A)** Schematic for tracing monosynaptic inputs to LC-NE neurons within male and female Dbh-Cre mice. **(B)** Timelines for experimental design. Co-packaged AAV helpers, lAAV-DIO-GT/RG was injected into the LC of both male and female Dbh-Cre mice (day 0). Three weeks later (day 21), genetically modified RV (EnvA-SADΔG-DsRed) was injected into same brain region for nine days’ expression (day 30) before sacrifice. **(C)** Representative confocal images showing starter cells in the injection site (LC) of male **(upper panel)** and female **(lower panel)** Dbh-Cre mouse brain sample. Left panel, low-magnification images; right panel, enlargement of white box regions. Locus coeruleus noradrenergic starter cells (indicated by white arrowheads) were labeled in yellow with the merge of green (EGFP) and red signals (DsRed) for both groups. Brain outlines were depicted with assistance of DAPI staining. Scale bars, 100 μm. **(D)** Quantification of starter cells in LC within male and female mice (M refers to male and F refers to female hereafter). **(E)** Quantification of total input cells of LC-NE neurons across the whole brain within male and female mice. **(F)** Comparisons of convergent index (i.e., the ratio of input cells over starter cells) (Miyamichi et al., 2013) of LC-NE neurons in male and female mice. Each circle represents one animal. Data are presented as the mean ± s.e.m. B, barrington’ nucleus; CBX, cerebellar cortex; LC, locus coeruleus; LDT, laterodorsal tegmental nucleus; PB, parabrachial nucleus; V4, fourth ventricle.

To examine the differences between the input circuitry of male and female mice, we utilized the well-established cell-type-specific RV-mediated monosynaptic tracing systems to demonstrate brain-wide presynaptic inputs of LC-NE neurons in both groups (Watabe-Uchida et al., 2012; Miyamichi et al., 2013; Schwarz et al., 2015). In this strategy, three essential elements, a fluorescent protein to denote starter cells, a TVA receptor for the entrance of genetically modified RV, and glycoprotein for the resembling of infectious RV particles, were delivered by two Cre-inducible recombinant AAV (rAAV) vectors. To eliminate the effect originated from the process of mixing two rAAVs, such as inaccurate regulation of mixing ratios and uneven distribution of virions within mixtures, we took advantage of the viral co-packaging strategy according to our previous method and used co-packaged lAAV-DIO-GT/RG as helper for monosynaptic tracing (see section “Materials and Methods”). To restrict starter cells to LC-NE neurons, we injected equal volumes of lAAV-DIO-GT/RG into the LC of male and female Dbh-Cre transgenic mice (Gong et al., 2007), followed by the injection of an equal volume of genetically modified EnvA-pseudotyped rabies virus (EnvA-SADΔG-DsRed) into the same location (Figures 1A,B). Supplementary Figure 2A showed that the starter cells identified by the co-expression of EGFP and DsRed had a similar distribution pattern in both male and female mice, ranging from −5.1 to −5.8 mm from the bregma in LC). However, the total numbers of the starter cells in male mice were 297 ± 29, which were 1.5 times higher than those in female mice (Figures 1C,D) (179 ± 22; mean ± s.e.m.; *n* = 4 mice for each group). We next counted DsRed-labeled input cells (note that DsRed signals adjacent to the injection site were excluded from the final count due to background labeling, see below) across the whole brain in all animals. We found that male mice had a slightly higher number of input cells (47,002 ± 1,383 for male mice and 39,496 ± 3,402 for female mice) than female mice (Figure 1E). However, the ratio of the total direct input cells to the starting cells was 160 on average in male mice and 213 for female mice (Figure 1F), indicating that female LC-NE neurons tend to receive more inputs.

### Whole-Brain Direct Inputs to Male and Female LC-NE Neurons

**FIGURE 2:**
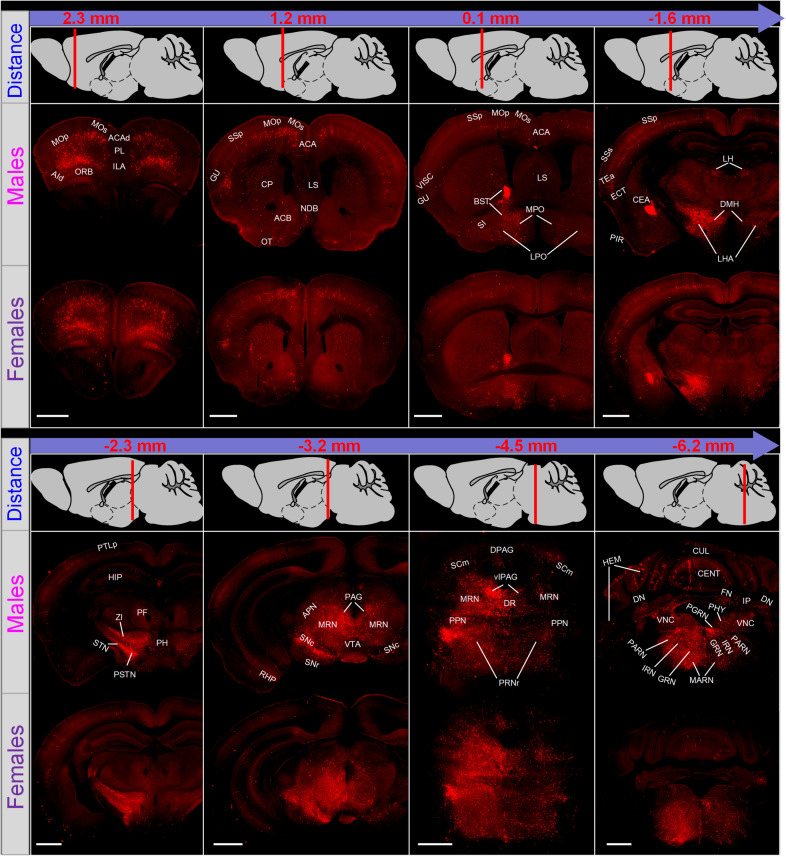
Overview of whole brain input to LC-NE neurons in male and female mice. Eight representative coronal sections along the anterior–posterior axis were displayed to show the distribution of presynaptic partners of LC-NE neurons in male **(middle)** and female **(lower)** mice. Distance from the bregma was shown with a sketch of brain on sagittal plane **(top)**. Scale bar, 500 μm. ACA, anterior cingulate area; ACAd, anterior cingulate area, dorsal part; ACB, nucleus accumbens; AId, agranular insular area, dorsal part; APN, anterior pretectal nucleus; BST, bed nuclei of the stria terminalis; CEA, central amygdalar nucleus; CENT, central lobule; CP, caudoputamen; CUL, culmen; DPAG, dorsal periaqueductal gray; DN, dentate nucleus; DR, dorsal raphe nucleus; ECT, ectorhinal area; FN, fastigial nucleus; GRN, gigantocellular reticular nucleus; GU, gustatory areas; HEM, hemispheric regions; HIP, Hippocampal region; ILA, infralimbic area; IP, interposed nucleus; IRN, intermediate reticular nucleus; LH, lateral habenula; LHA, lateral hypothalamic area; LPO, lateral preoptic area; LS, lateral septal nucleus; MARN, magnocellular reticular nucleus; MOp, primary motor area; MOs, secondary motor area; MPO, medial preoptic area; MRN, midbrain reticular nucleus; NDB, diagonal band nucleus; ORB, orbital area; OT, olfactory tubercle; PAG, periaqueductal gray; PARN, parvicellular reticular nucleus; PF, parafascicular nucleus; PGRN, paragigantocellular reticular nucleus; PH, posterior hypothalamic nucleus; PHY, perihypoglossal nuclei; PIR, piriform area; PL, prelimbic area; PPN, pedunculopontine nucleus; PRNr, pontine reticular nucleus; PSTN, parasubthalamic nucleus; PTLp, posterior parietal association areas; RHP, retrohippocampal region; SCm, superior colliculus motor related; SI, substantia innominate; SNc, substantia nigra, compact part; SNr, substantia nigra reticular part; SSp, primary somatosensory area; SSs, supplemental somatosensory area; STN, subthalamic nucleus; TEa, temporal association areas; VISC, visceral area; VLPAG, ventrolateral periaqueductal gray; VTA, ventral tegmental area; ZI, zona incerta.

To trace long-range monosynaptic inputs to male and female LC-NE neurons, we imaged the coronal sections across the whole brain (ranging from +3.0 to −6.36 mm). In both groups, an average of 97 coronal sections were obtained (96.8 ± 0.5 coronal sections for male mice and 96.5 ± 1.4 for female). Overall, LC-NE neurons of the two groups shared similar input sources and exhibited a bilateral symmetrical manner for most brain regions (Figure 2). For example, they received inputs from nearly all cortical regions, predominantly in somatomotor areas (MO, primary and secondary), somatosensory areas (SS, primary and secondary) and prefrontal areas (medial and orbitofrontal). The densest inputs in both groups were uniformly found in several subcortical areas, including the BST in the pallidum, the central amygdala nucleus (CEA) in the striatum, the lateral hypothalamic area (LHA) and zona incerta (ZI) in the hypothalamus, the periaqueductal gray (PAG), MRN and superior colliculus, motor related (SCm) in the midbrain, the medulla, and the PRNr and pontine reticular nucleus, caudal part (PRNc) in pons, all with a bilateral symmetrical manner. Furthermore, as a member of the modulatory families, LC-NE neurons received inputs from all three major modulatory systems, such as the cholinergic neuron-nested medial septal complex (MSC, including medial septal complex and diagonal band nucleus), dopaminergic neuron-nested ventral tegmental area (VTA) and substantia nigra, compact part (SNc), and serotonergic neuron-nested dorsal nucleus raphe (DR).

### Strategies for Brain-Wide Analysis of Inputs to Male and Female LC-NE Neurons

**FIGURE 3:**
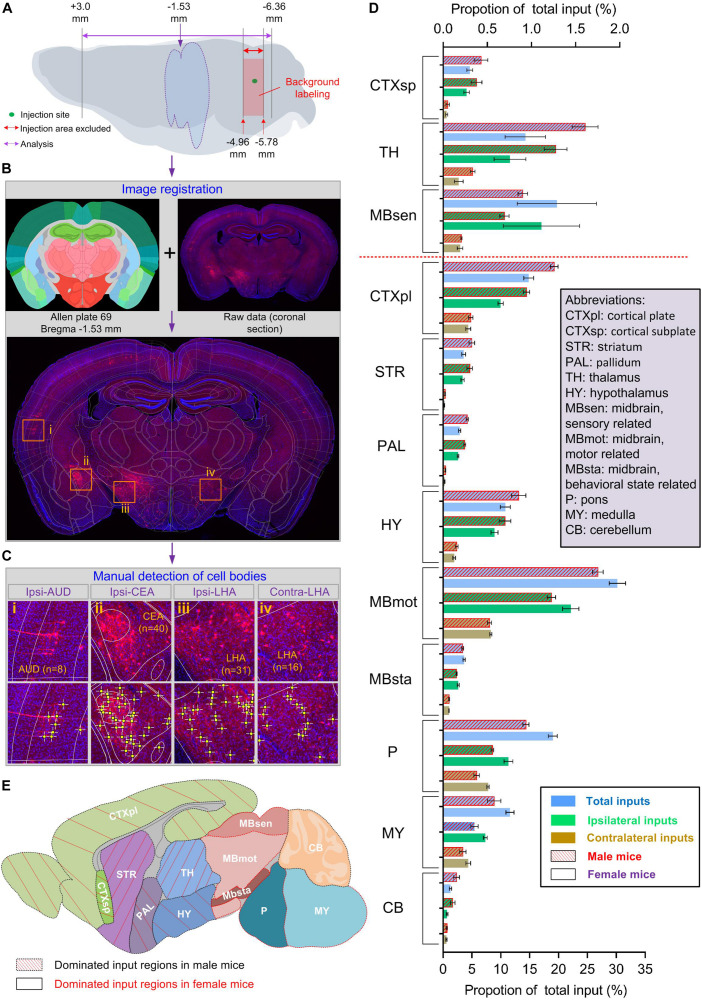
LC-NE neurons in both sexes received differential input proportions. **(A–C)** Procedures for data analysis. **(A)** Schematic of brain regions used for analysis. The imaged coronal sections ranging from +3.0 to −6.36 mm from bregma were used in each individual animal. Injection sites with background labeling (−4.96 to −5.78 mm from bregma) were excluded from the analysis. **(B)** Schematic of registrations. Each dual-color raw image (e.g., a coronal section at −1.53 mm from bregma; blue: DAPI staining signals and red: DsRed-positive input signals) were registered to the corresponding plate (e.g., plate 69) of the standard Allen Mouse Brain Reference Atlas. **(C)** Schematic of manual cell counting. Within each registered coronal section, cell bodies located in different sub-regions were identified and the total numbers were counted manually. Four box regions (i–iv) were enlarged from **(B)** to show cell counting in different anatomical regions: ipsi-AUD (i, *n* = 8 cells), ipsi-CEA (ii, *n* = 40 cells), ipsi-LHA (iii, *n* = 31 cells), and contra-LHA (iv, *n* = 31 cells). **(D)** Quantitative analysis of the proportion of total, ipsilateral, and contralateral inputs in 12 major brain regions for LC-NE neurons in both sexes. The red dotted line separated three regions with sparse inputs from nine regions with moderate and dense inputs. Data are presented as the mean ± s.e.m., *n* = 4 mice for each group. **(E)** Summary of distribution of brain regions with dominated inputs to male and female LC-NE neurons. Abbreviations: AUD, auditory areas; CEA, central amygdala nucleus; LHA, lateral hypothalamic area.

To investigate the sex-related different inputs of LC-NE neurons more comprehensively, we aimed to generate the whole-brain input atlas of LC-NE neurons in both sexes to obtain the information of: (i) the precise anatomical localization of input signals and (ii) the number of input neurons in each anatomical area. To address these issues, we initially registered each imaged section within an individual whole-brain data set to the standard Allen Mouse Brain Reference Atlas with the assistance of landmarks afforded by DAPI staining (Figure 3B and Supplementary Figure 3). We further used manual counting as ground truths to identify DsRed-positive input signals (see Supplementary Figure 1B and online Methods for more details of manual identifications) and summarized the total number of input neurons in each anatomical area (Figure 3C and Supplementary Figure 1C). Similar to previous studies (Watabe-Uchida et al., 2012; Miyamichi et al., 2013; Schwarz et al., 2015; Do et al., 2016), we excluded the counting of the injection site and similar adjacent areas from the final analysis for both male and female groups due to the background labeling resulting from the leakage of TVA (Figure 3A and Supplementary Figure 1D).

**FIGURE 4:**
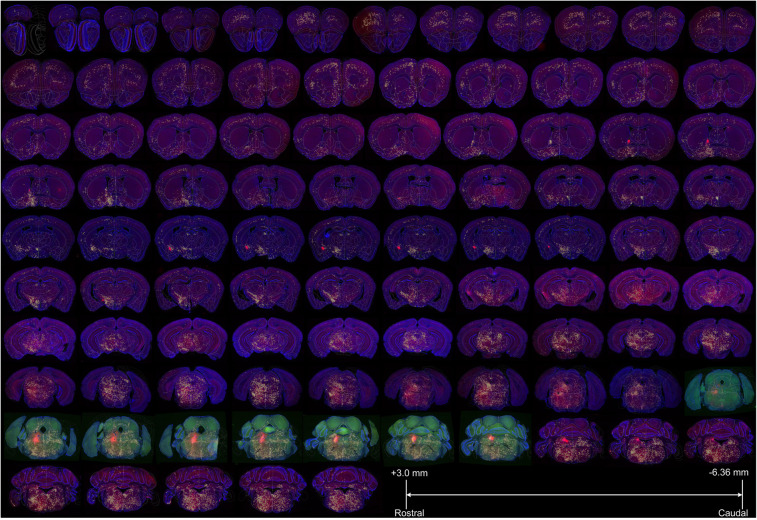
The registered whole-brain data set of input sources of LC-NE neurons. A representative female mouse brain (F3) with a total of 97 coronal sections (with the range of +3.0 to −6.36 mm from bregma) were displayed sequentially to show the brain-wide distribution of presynaptic inputs to LC-NE neurons after the registration to the standard Allen Mouse Brain Reference Atlas. Yellow across within each section indicated the manually identified input cells and eight sections with green signals indicated injection site areas. Blue: DAPI staining signals, red: DsRed-positive input signals and green: EGFP.

The criterion we adopted for anatomical classifications were mainly based on specifications introduced by the Allen Brain Atlas (sunburst mode), similar to previous studies (Pollak Dorocic et al., 2014; Do et al., 2016; Zhang et al., 2016). After registrations, the whole brain was divided into 257 anatomical regions belonging to 12 major regions (Figure 4 and Supplementary Figure 4). To make the quantitative comparisons faithfully, we normalized the input cells in each anatomical region over the total number of input cells of each individual animal. Because no inputs were found to be from only one brain side across these regions, we compared both ipsilateral and contralateral proportions of inputs at different levels. These analyses include the comparisons in: (i) Major regions (gross level, mainly 4th grades in Allen Brain Atlas); (ii) Multiple sub-regions (moderate level, mainly 5th grades in Allen Brain Atlas); and (iii) Specific subsets of nuclei within sub-regions (fine-scale level, mainly 6th grades in Allen Brain Atlas).

### Male and Female Mice Showed Differential Proportion of Inputs to LC-NE Neurons

We first examined inputs of LC-NE neurons on a gross level. The midbrain, motor-related (MBmot) area provided the largest inputs in both groups among 12 major regions, with an average 30.2% out of total inputs for females, a little higher than males (26.8%). Similar to MBmot, we found discriminated distributions of proportion of inputs in the other 11 major regions. Specifically, inputs were dominant in the cerebrum, interbrain, and cerebellum (CB), the former two including the cortical plate (CTXpl), cortical subplate (CTXsp), striatum (STR), pallidum (PAL), thalamus (TH), and hypothalamus (HY) in males; whereas inputs were dominant in the midbrain and hindbrain, which include the sensory-/motor-/behavioral state-related midbrain part (MBsen/MBmot/MBsta), pons (P), and medulla (MY) in females (Figures 3D,E). For instance, inputs from CTXpl (averaged at 19.3%) was the second largest in males, whereas it was only averaged at 14.8% in females, which was smaller even than pons (averaged 19.0%), far from comparable with MBmot (Figure 3D). These results collectively indicated that male mice were distinguishable from females with differential proportion of inputs to LC-NE neurons, though they weer received from the same set of brain regions.

**FIGURE 5:**
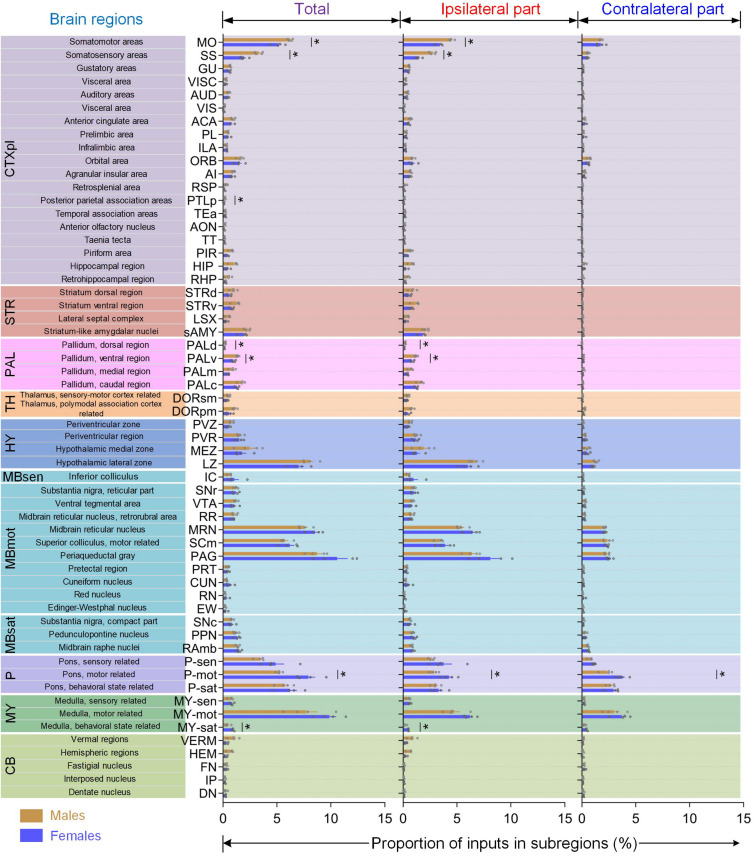
Quantitative comparisons of brain-wide monosynaptic inputs to LC-NE neurons in male and female mice. 59 sub-regions in male and female mice with averaged total proportion of input >0.1% **(left)** were shown along with the comparisons of inputs from ipsilateral **(middle)** and contralateral hemisphere **(right)**. Data are presented as the mean ± s.e.m., *n* = 4 mice for each group. Each gray circle represents the data of one mouse. Brain regions with a statistical difference were marked using Mann–Whitney U test, *P < 0.05.

We next examined the differences between inputs to male and female LC-NE neurons in specific sub-regions of the 12 major brain regions. Among 257 distinct anatomical regions, 123 were identified with proportion of inputs >0.1% (over 40 cells per region) and were used for further direct comparisons (Figure 5). We initially examined the differences between two sexes in areas (the cerebrum, interbrain, and cerebellum) where inputs were dominant in males. Overall, the sub-regions with substantial differentials of proportion of inputs between the two sexes among these male-dominant input areas were the hippocampal region (HPF), TH, cerebellar cortex (CBX), specific parts of the cortex, STR, PAL, and HY.

Among diverse cortical sub-regions (Figure 5, gray part), the MO and SS provided major inputs to LC-NE neurons (averaged 9.57% out of 16.36% for male and 7.07% out of 12.8% for female). Furthermore, both the MO and SS showed significantly differential proportions of inputs (male versus female: 6.25 ± 0.11% and 5.14 ± 0.29% for MO, 3.32 ± 0.19% and 1.93 ± 0.18% for SS; *P* = 0.0286, Mann–Whitney test), which were derived from ipsilateral hemispheres rather than contralateral hemisphere (take SS as an example, ipsilateral: 2.76 ± 0.15% versus 1.44 ± 0.13% for male and female respectively; contralateral: 0.57 ± 0.06% versus 0.51 ± 0.05% for male and female respectively). Across the remaining cortical sub-regions, we observed that visual (VIS), retrosplenial (RSP), and posterior parietal association areas (PTLp) provided more than 2-fold total, ipsilateral, and contralateral proportion of inputs to male mice than females. The other sub-regions, including several subdivisions in medial prefrontal areas (anterior cingulate area, ACA; prelimbic area, PL and infralimbic area, ILA), the orbital area (ORB), agranular insular area (AI), gustatory areas, visceral area, auditory areas, and temporal association areas, all shared similar proportion of inputs between male and female mice. Moreover, we also observed that no significant statistical differences were found in the sub-regions of HPF, the hippocampal formation (HIP), and the parahippocampal region (RHP), however, there was a prominent trend toward projecting to male mice, with over 2-fold difference in total, ipsilateral, and contralateral inputs when compared with female mice (Supplementary Figure 5A).

In accordance with previous studies (Schwarz et al., 2015), we also found substantial inputs in the CBX and cerebellar nuclei (CBN) in both sexes which extended along the anterior–posterior axis caudally (Supplementary Figure 5C). However, in the sub-regions of the CBX, vermal regions (VERM), and hemispheric regions (HEM) provided preferentially larger proportions of inputs to male mice, with nearly 3-fold of that to females (Figure 5, light green part and Supplementary Figure S5B). In contrast, three sub-regions in the CBN, fastigial nucleus (FN), interposed nucleus (IP), and dentate nucleus (DN), all showed nearly identical proportion of inputs between the two sexes (Figure 5, light green part).

When dividing STR into four sub-regions (Figure 5, brown part), we found that striatum-like amygdala nuclei (sAMY), which accounted for the largest striatal inputs, provided similar proportions in male (averaged 2.26%) and female mice (averaged 1.90%). The other three sub-regions, from striatum ventral region (STRv) to striatum dorsal region (STRd), and lateral septal complex (LSX), progressively showed a prominent trend toward providing inputs to male mice for not only total parts but ipsilateral and contralateral hemispheres. In PAL, both of its dorsal (PALd) and ventral sub-regions (PALv) showed significantly differential proportions of inputs between male and female mice (*P* = 0.0286, Mann–Whitney test), which were also derived from the ipsilateral hemisphere, similar to MO and SS in cortical regions (Figure 5, pink part). In HY, only the periventricular zone (PVZ) showed a prominent trend toward providing inputs to male mice, whereas the other three sub-regions, periventricular region (PVR), hypothalamic medial zone (MEZ), and hypothalamic lateral zone (LZ), all contained similar proportions in male (averaged 4.02%) and female mice (averaged 3.42%; Figure 5, blue part).

We also examined the differences between the two sexes in the midbrain and interbrain where inputs were dominant in females. Overall, the differentials in these female-dominant input areas were not as apparent as in males. In MBmot (Figure 5, cyan part), most sub-regions showed a similar proportion of input between male and female mice, such as substantia nigra, reticular part (SNr), VTA, PAG, MRN, and SCm, except for two sub-regions, cuneiform nucleus (CUN) and red nucleus (RN), both of which showed a prominent trend toward providing inputs to female mice. Among three sub-regions in pons (Figure 5, purple part), the motor-related region (P-mot) which makes up the second largest inputs to behavioral state regions (P-sat), showed a significant statistical difference of total inputs, which was also derived from the ipsilateral hemisphere (*P* = 0.0286, Mann–Whitney test), whereas the latter showed a similar proportion. Similarly, behavioral state regions (MY-sat), which contributed the fewest proportion of inputs to the medulla, also showed significant statistical difference of both total and ipsilateral inputs (*P* = 0.0286, Mann–Whitney test) between the two sexes with over 2-fold more inputs to females.

In summary, mice of two sexes showed differential proportion of inputs to LC-NE neurons not only in major brain regions but also in diverse sub-regions.

### Sex-Related Differential Inputs to LC-NE Neurons Originated From Specific Nuclei

**FIGURE 6:**
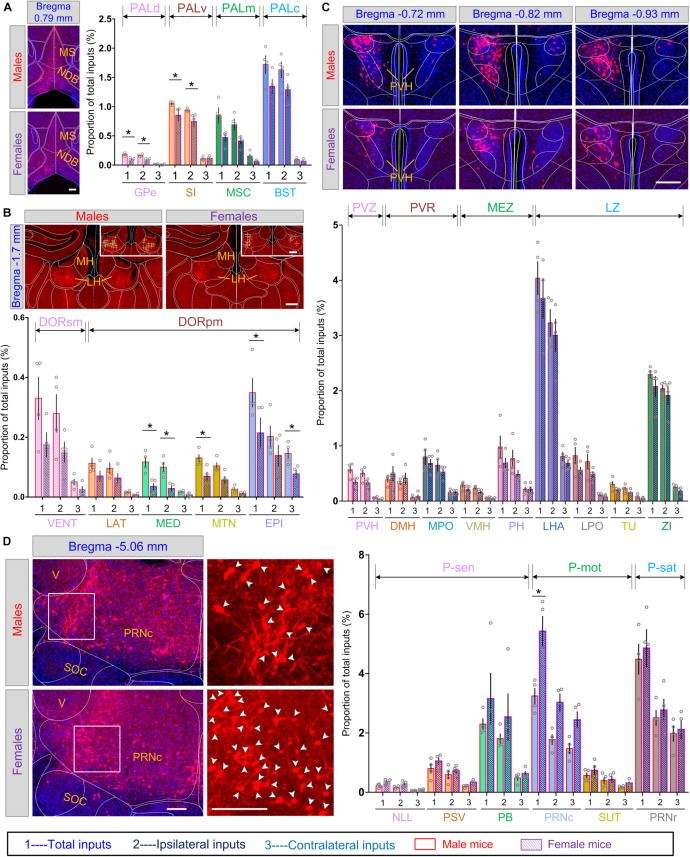
Comparisons of monosynaptic inputs to LC-NE neurons among diverse nuclei of male and female mice. Scale bar, 100 μm. **(A)** Left panel, representative images showing DsRed-labeled input cells in MS/NDB of male and female mice. Right panel, quantitative comparisons of the proportion of inputs within several nuclei of pallidum between male and female mice. Scale bars, 100 μm. **(B)** Upper panel, representative images showing DsRed-labeled input cells in the epithalamus of male and female mice. Lower panel, quantitative comparisons of the proportion of inputs within several nuclei of the thalamus between male and female mice. Scale bars, 100 μm. **(C)** Upper panel, three adjacent coronal sections showing the distribution of DsRed-labeled input cells in the PVH targeting male and female LC-NE neurons. Lower panel, quantitative comparisons of the proportion of inputs within several nuclei of the hypothalamus between male and female mice. Scale bars, 100 μm. **(D)** Left panel, representative images showing DsRed-labeled input cells in PRNc of male and female mice. Right panel, quantitative comparisons of the proportion of inputs within several nuclei of pons between male and female mice. Scale bars, 100 μm. Data are presented as the mean ± s.e.m., *n* = 4 mice for each group. Each gray circle represents the data of one mouse. Brain regions with statistical difference were marked using Mann–Whitney U test, *P < 0.05. Abbreviations: BST, bed nuclei of the stria terminalis; DMH, dorsomedial nucleus of the hypothalamus; DORpm, thalamus, polymodal association cortex related; DORsm, thalamus, sensory-motor cortex related; EPI, epithalamus; GPe, globus pallidus, external segment; LAT, lateral group of dorsal thalamus; LH, lateral habenula; LHA, lateral hypothalamic area; LPO, lateral preoptic area; LZ, hypothalamic lateral zone; MED, medial group of dorsal thalamus; MEZ, hypothalamic medial zone; MH, medial habenula; MPO, medial preoptic area; MS, medial septal complex; MSC, medial septal complex; MTN, midline group of dorsal thalamus; NDB, diagonal band nucleus; NLL, nucleus of the lateral lemniscus; PALc, pallidum, caudal region; PALd, pallidum, dorsal region; PALm, pallidum, medial region; PALv, pallidum, ventral region; PB, parabrachial nucleus; PH, posterior hypothalamic nucleus; P-mot, pons, motor related; PRNc, pontine reticular nucleus, caudal part; PRNr, pontine reticular nucleus; P-sat, pons, behavioral state related; P-sen, pons, sensory related; PSV, principal sensory nucleus of the trigeminal; PVH, paraventricular hypothalamic nucleus; PVR, periventricular region; PVZ, periventricular zone; SI, substantia innominate; SOC, superior olivary complex; SUT, supratrigeminal nucleus; TU, tuberal nucleus; V, motor nucleus of trigeminal; VENT, ventral group of dorsal thalamus; ZI, zona incerta.

The above data showed that at some of the gross brain regions, and at some of the sub-regions within the corresponding gross regions, the inputs were sexually differentiated. We suspect that this might be true at an even finer level, that is, specific subsets of nuclei within the corresponding sub-regions. To gain further insight into the contributions and distributions of these distinct nuclei, we therefore investigated input circuitry to LC-NE neurons in more detail. In the pallidum, Globus pallidus, external segment (GPe), substantia innominate (SI), MSC, and BST took the majority of inputs in the dorsal, ventral, medial, and caudal regions of PAL, respectively. Among these four subdivisions, GPe and MSC in males contained nearly 2-fold more proportion of inputs and showed significant statistical difference of both total and ipsilateral inputs (*P* = 0.0286, Mann–Whitney test) between the two sexes, whereas SI and BST contained similar inputs for both groups (Figure 6A, right panel). Further, within MSC, inputs for females were located mainly in one of the two nuclei, NDB, whereas in males, inputs were distributed within both MS and NDB (Figure 6A, left panel).

Quantitative analysis across subsets of nuclei within the thalamus revealed that the epithalamus (EPI) and ventral group of dorsal thalamus (VENT) accounted for the largest inputs in DORsm and DORpm, respectively. We found that subdivisions in DORsm (i.e., lateral, medial, and midline groups of dorsal thalamus and EPI) and DORpm (VENT, the sole input region), all showed averaged 2-fold more proportion of inputs in males, both from ipsilateral and contralateral hemispheres, though statistical difference was not significant (Figure 6B, lower panel). Furthermore, sex-related differential inputs in EPI were derived from lateral habenula (LH), the critical region involved in depression (Li et al., 2013), since far fewer inputs were found in the medial habenula (MH), the other main nucleus in EPI (Figure 6B, upper panel). Preferential inputs were also observed among multiple nuclei in the hypothalamus. Paraventricular hypothalamic nucleus (PVH) in the PVZ, a critical brain region for homeostasis (Li et al., 2019), showed a prominent trend toward providing inputs to male mice (Figure 6C, upper panel). In contrast, LHA and ZI, which accounted for the largest proportions of inputs in the hypothalamus, shared a similar proportion of inputs between the two sexes (Figure 6C, lower panel). Furthermore, we found that the proportion of inputs differentiated in sexes along dorsal and ventral axis in the hypothalamus. For instance, the dorsomedial nucleus of the hypothalamus (DMH) within the PVR acted as one of the few nuclei across the interbrain with less proportion of inputs in male (0.38 ± 0.05% for male versus 0.49 ± 0.14% for female). However, the ventromedial nucleus of the hypothalamus (VMH) within the hypothalamic MEZ showed more proportion of inputs in males than females (0.29 ± 0.04% for male versus 0.21 ± 0.03% for female) (Figure 6C, lower panel).

In pons, PB, PRNr, and PRNc made up the majority of inputs in sensory related, behavior state related, and motor related pons, respectively. We observed more significant differential total inputs in PRNc between the two sexes (5.43 ± 0.52% for female versus 3.25 ± 0.27% for male; *P* = 0.0286, Mann–Whitney test) when compared with PRNr, which showed similar inputs (4.86 ± 0.64% for female versus 4.49 ± 0.52% for male) (Figure 6D).

In summary, male and female LC-NE neurons shared similar input regions, but with differentiated inputs from some of the sub-regions, and further, from some of the nuclei within the corresponding sub-regions.

**FIGURE 7:**
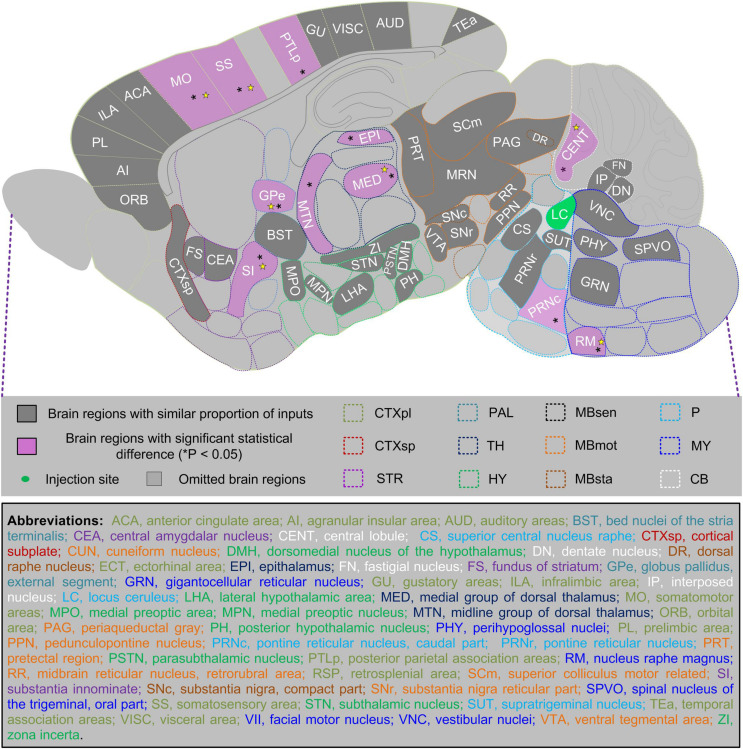
Summarized sex differences-related whole-brain input map of LC-NE neurons. We scanned through 123 anatomical regions with a total proportion of input over 0.1% and compared proportion of total, ipsilateral, and contralateral inputs in male and female mice (*n* = 4 mice for each). Among 52 anatomical regions demonstrated in the sagittal plane, 41 of them showed a similar proportion of total inputs (indicated by gray color), whereas 11 of them showed significant statistical difference (*P < 0.05) between proportion of total inputs of two sexes (indicated by purple color) and over half of them (*N* = 7) also showed such differences in ipsilateral hemispheres (indicated by yellow star). Mann–Whitney U test.

Based on these observations, we further compared proportions of total, ipsilateral, and contralateral inputs in both sexes among 123 anatomical regions (with total proportion of input over 0.1%). We found that 41 of them, which made up over one-third of the anatomical regions, showed a similar proportion of inputs in male and female mice. Furthermore, we identified 11 anatomical regions with significant statistical differences of total inputs between the two sexes, and seven of them also showed such differences in ipsilateral hemispheres, with only one region showing such differences in the contralateral hemisphere (Figure 7). These data, combined with comparisons in 12 major brain regions, collectively constitute sex-related differences in a whole-brain input map of LC-NE neurons.

## Discussion

Our study provided the brain-wide quantitative analysis of direct inputs to LC-NE neurons in male and female mice and generated the sex-related differential whole-brain input atlas for the first time (to the best of our knowledge). For 257 anatomical areas covering the whole brain, proportions of inputs at different regional, sub-regional, and nuclear levels were analyzed. We found that LC-NE neurons of two sexes shared an overall similarity of input patterns, but with differential inputs from major brain regions and diverse sub-regions (Figures 3,5). Inputs to male LC-NE neurons were dominant in the cerebrum, interbrain, and cerebellum, whereas inputs to female neurons were dominant in the midbrain and hindbrain (Figures 3D,E). Specific nuclei within sub-regions contributed to overall sex-related differential inputs (Figure 6). Among a totaled 123 anatomical regions with proportion of inputs >0.1%, we found that 11 of them showed significant statistical differences of total inputs between male and female mice, and seven of them also showed such differences in ipsilateral hemispheres (Figure 7).

With brain-wide quantitative analysis of input sources to LC-NE neurons, we identified multiple sexually differentiated anatomical brain regions, including the hippocampus, MSC in basal forebrain, thalamus, PVH in hypothalamus, and cerebellar cortex. The findings are consistent with previous studies in rats showing that somal and dendritic differences exist within different sexes (Pinos et al., 2001; Garcia-Falgueras et al., 2005; Bangasser et al., 2011). For the hippocampus, its connections with LC play important roles in the regulation of learning/memory and different performances. The differential connections may offer a structural basis to account for the better spatial cognitive capability in males (Shah et al., 2004; Choleris et al., 2018). Locus coeruleus noradrenaline has been proven to be involved in Alzheimer’s disease (AD) both experimentally and clinically, involving the circuits formed among several brain regions, such as the hippocampus and basal forebrain (Jacobs et al., 2013). We found that males contained more averaged total inputs from the HPF and MSC (Figure 6A and Supplementary Figure 5A), which might help to reduce the risk of the prevalence of AD in males (Ungar et al., 2014). Several brain regions, including (ACB) in the striatum ventral region (STRv), LH in EPI, and ventral subiculum in RHP, are involved in depression (Zagni et al., 2016; Labonté et al., 2017). We observed a more total proportion of inputs receiving from the ACB, LH, and RHP for males (Figures 5,6B), providing a basis for the clinically observed fact that women have a higher prevalence of developing depression (Green et al., 2019). Thus, our study provides hints for further studies related to LC-NE neurons on a variety of functions. It is surprising that several nuclei that send dense inputs to LC-NE neurons, such as BST, CeA, LHA, and PAG (Figure 5), did not have apparent differences between sexes, although these regions are important and sexually dimorphic in anxiety, fear, depression, and pain. Other mechanisms, such as different proportions of excitatory and inhibitory neurons, are involved.

We also compared the direct input networks of LC-NE neurons in both sexes with careful considerations of age (2-month old), weight (21–23 g), viruses (co-packaged rAAV helper), and data analysis (comparisons within the same area ranging from Bregma +3.0 to −6.36 mm and exclusion of the areas adjacent to the injection site). However, menstrual cycle was not taken into consideration, which might affect the normal conditions of female animals (Bayer et al., 2013). Though Dbh-Cre transgenic mice of two sexes with similar body weight were used in our study, it is worth noting that transgenic and wild-type mice shared similar differences in body weight between males and females, as indicated by previous studies (Somerville et al., 2004; Lee et al., 2018). Moreover, we registered and counted the input cells across the whole brain manually in this study. Though high accuracy could be reached, it is inevitably time-consuming and labor-intensive. Therefore, we only made whole-brain analysis among eight male and female mice. It is undoubtable that more reliable conclusions could be drawn when more samples were involved, particularly for several brain regions such as the HPF and CBX, since statistical differences between two sexes were not significant though with 2–3 fold enrichment of inputs to male mice. It is worthy to note that among the 123 brain regions proportion of inputs >0.1%, only 11 brain regions showed significant statistical differences in total inputs, which could also be due to random fluctuations. Moreover, considering the possible variability in injection site, labeling efficiency, and individual differences, the power of statistical significance analysis could be limited for elucidating potentially subtle difference between sexes. Therefore, independent methods (e.g., channelrhodopsin (ChR2)-assisted circuit mapping) are still in urgent need to solidify our conclusions. Additionally, the background labeling of the rAAV helpers (AAV-GT) forced us to exclude the data from the injection site (including part of CUN, PB, and SUT); studies focused on the local inputs should take advantages of other complementary strategies, such as genetically modified TVA (TVA^66*T*^) (Miyamichi et al., 2013). Further, the data here are only the direct input networks, a more comprehensive understanding can only be achieved when the directed output networks are also available. Additionally, we should be aware that, similar to the conclusions drawn from the study of sex hormones (Arnold, 2004; Davies and Wilkinson, 2006), the differences on input circuitry solely fail to elucidate the whole mechanisms for sex differences. Regardless, the results from this study could provide suggestions or cautions to many different researches. For example, animal sex should be taken into consideration when regions with substantially different inputs to LC-NE are the targets of the planned projects.

In summary, our study not only provides the structural basis for our understanding of sex differences in LC-NE neurons at the circuitry level but also provides clues for related further functional studies. Further, the strategies employed in our study offer a paradigm applicable for the input networks in other brain regions or neuron types. Therefore, our data and strategies should be helpful for the neuroscience community for a broad range of functional and structural research.

## Data Availability Statement

All datasets generated for this study are included in the article/Supplementary Material.

## Ethics Statement

The animal study was reviewed and approved by Institutional Animal Ethics Committee of Huazhong University of Science and Technology (HUST).

## Author Contributions

Y-HZ conceived the project. PS, JW, and MZ performed the virus injection experiments. PS performed the imaging with the assistance of YM and performed the whole-brain registrations. PS, JW, YM, YW, XD, and YM performed manual counting of the input cells. PS and JW analyzed the data. FX provided helpful advice for manuscript. Y-HZ, PS, and JW wrote the manuscript with discussion and improvements from all authors. All authors contributed to the article and approved the submitted version.

## Conflict of Interest

The authors declare that the research was conducted in the absence of any commercial or financial relationships that could be construed as a potential conflict of interest.

## Footnotes

1 https://www.biorxiv.org/content/10.1101/705772v1

2 http://atlas.brain-map.org/
