# Supplementary material for: Sex-Related Differential Whole-Brain Input Atlas of Locus Coeruleus Noradrenaline Neurons
Source: Front Neural Circuits. 2020 Sep 23;14:53. doi: 10.3389/fncir.2020.00053 (PMC7541090; doi:10.3389/fncir.2020.00053)
Supplement: Supplementary file 1 [file Data_Sheet_1.PDF]

Sex-related Differential Whole-brain Input Atlas of Locus Coeruleus Noradrenaline Neurons

Pei Sun[1,4,5], Junjun Wang[1,4,5], Meng Zhang[1,4], Xinxin Duan[1,4], Yunfei Wei[1,4], Fuqiang Xu[3], Yan Ma[2], Yu-Hui Zhang[1,4*]

[1]Britton Chance Center for Biomedical Photonics, Wuhan National Laboratory for Optoelectronics-Huazhong University of Science and Technology (HUST), Wuhan, Hubei 430074, China

[2]HUST-WHBC United Hematology Optical Imaging Center, Wuhan Blood Center (WHBC), Wuhan, Hubei 430030, China

[3]Centre for Brain Science, State Key Laboratory of Magnetic Resonance and Atomic Molecular Physics, Key Laboratory of Magnetic Resonance in Biological Systems, Wuhan Institute of Physics and Mathematics, CAS Centre for Excellence in Brain Science and Intelligence Technology, Chinese Academy of Sciences, Wuhan, Hubei 430071, China

[4]MoE Key Laboratory for Biomedical Photonics, School of Engineering Sciences, Huazhong University of Science and Technology, Wuhan, Hubei 430074, China

[5]These authors contributed equally to this work

Correspondence should be addressed to Y.-H. Z (email: zhangyh@mail.hust.edu.cn)

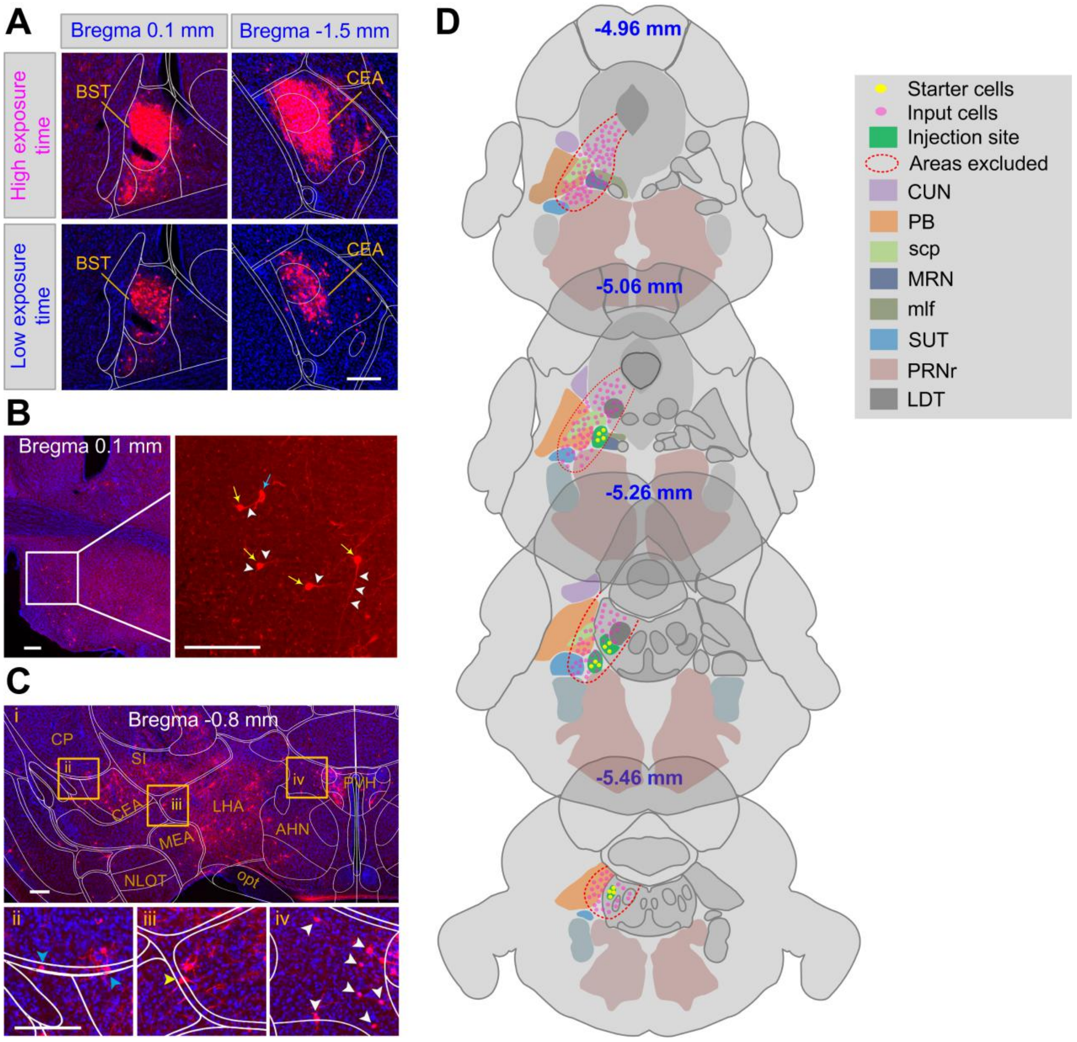

Supplementary Figure S1. Schematic of imaging, cell counting and data analysis.

(A) Representative images showing imaging with high and low exposure time in two brain regions (BST and CEA) with dense input signals. Scale bar, 100 μm.

(B) Representative image showing distinguishment between cell bodies (indicated by yellow arrows) and dendrites whose cell bodies were cut off (indicated by cyan arrows). White arrowheads indicated one or several apparent dendrites protruding from cell bodies to make them distinguishable from noise signals. Scale bars, 100 μm.

(C) Representative image showing the assignment of input cells in several specified cases: (i) Input cells located within space areas of two adjacent brain regions were regarded as a part of proximal regions. For instance, two input cells were located within borders of CP and CEA (indicated by yellow arrowheads), we assigned them to CEA due to more proximal to this region. (ii) Input cells with only part of cell bodies located in (indicated by cyan arrowheads) were also assigned to this brain region (LHA). (iii) Eight input cells adjacent to PVH were located in blank regions according to Allen Institute Mouse Brain Reference Atlas. We counted this type of cells separately. Scale bars, 100 μm.

(D) Schematic of four coronal sections showing the exclusion of background labeling areas in both male and female groups, which including injection site (LC) and LC-adjacent brain regions, such as a minority parts of PB, MRN, SUT and PRNr.

Abbreviations: AHN, anterior hypothalamic nucleus; BST, bed nuclei of the stria terminalis; CEA, central amygdalar nucleus; CP, caudoputamen; CUN, cuneiform nucleus; LDT, laterodorsal tegmental nucleus; LHA, lateral hypothalamic area; MEA, medial amygdalar nucleus; mlf, medial longitudinal fascicle; MRN, midbrain reticular nucleus; NLOT, nucleus of the lateral olfactory tract; opt, optic tract; PB, parabigeminal nucleus; PRNr, pontine reticular nucleus; PVH, paraventricular hypothalamic nucleus; scp, superior cerebelar peduncles; SI, substantia innominate; SUT, supratrigeminal nucleus.

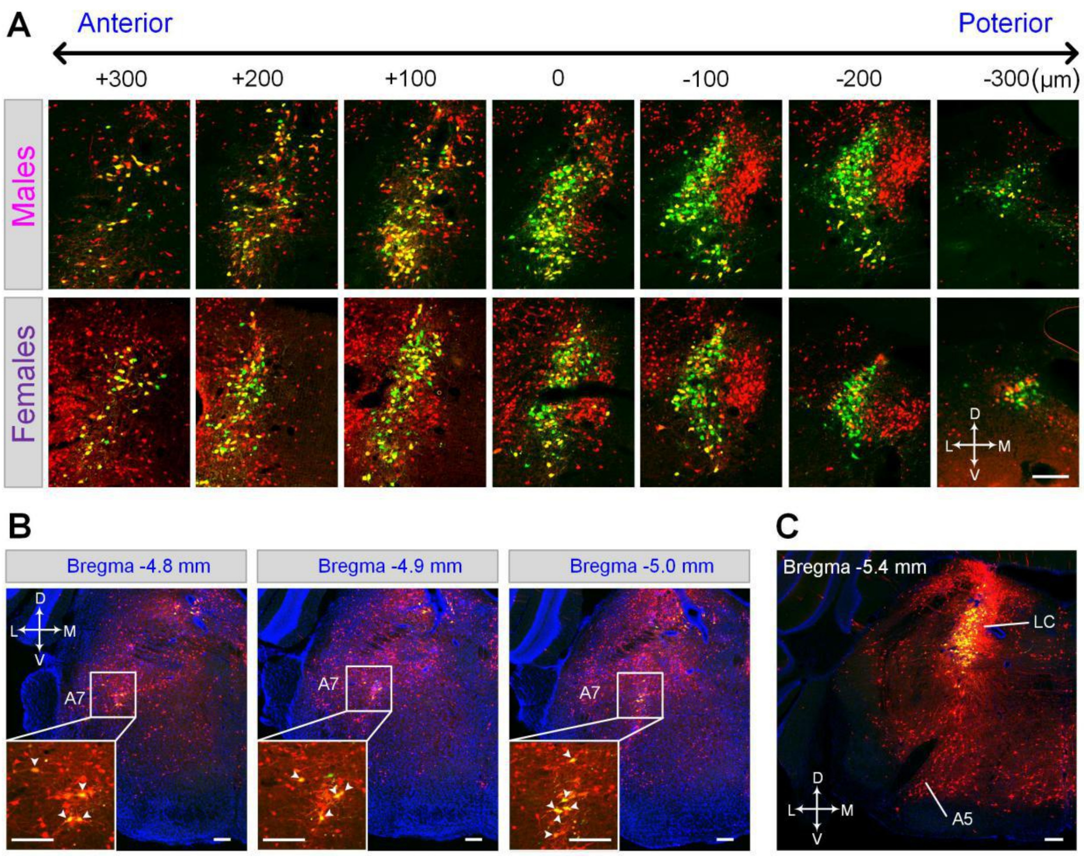

Supplementary Figure S2. Distributions of the starter cells in male and female Dbh-Cre transgenic mice.

(A) Seven representative images along the anterior-posterior axis (ranging from -5.1 to -5.8 mm from the bregma) were sequentially displayed to show the distributions of the starter cells in LC in male (upper panel) and female (lower panel) mice. Scale bars, 100 μm.

(B) Three representative images along the anterior-posterior axis (ranging from -4.8 to -5.0 mm from the bregma) were arrayed sequentially to show the distributions of the starter cells in A7. Scale bars, 100 μm.

(C) No starter cells were found in A5. Scale bars, 100 μm.

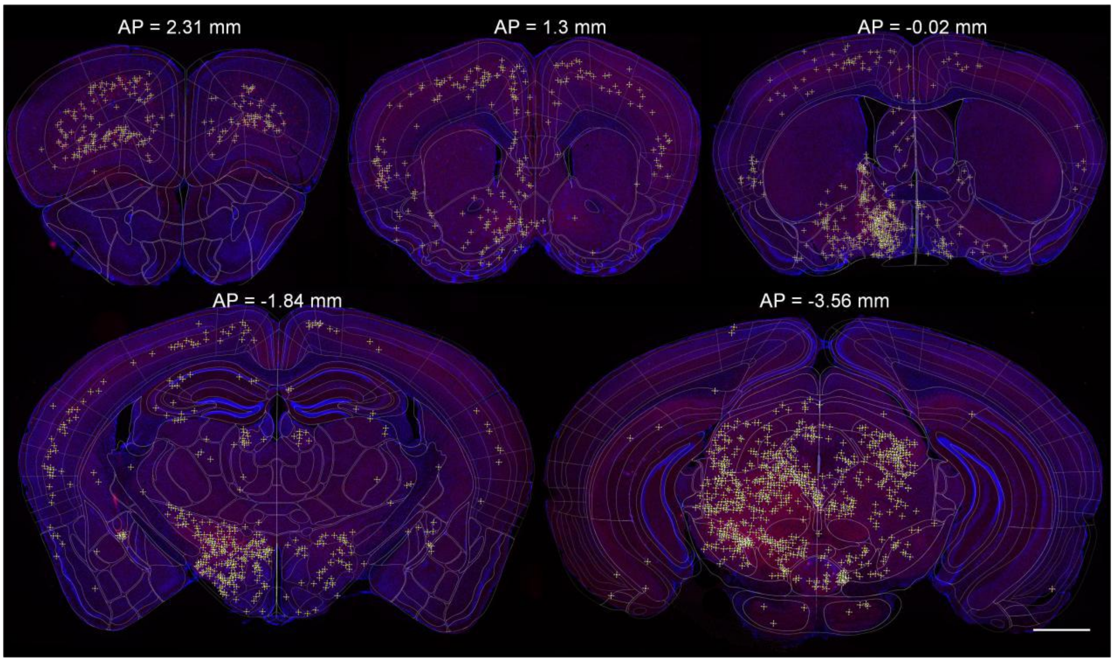

Supplementary Figure S3. Enlargements of five representative sections in Figure 4 showing the results of the registration. Scale bars, 100 μm.

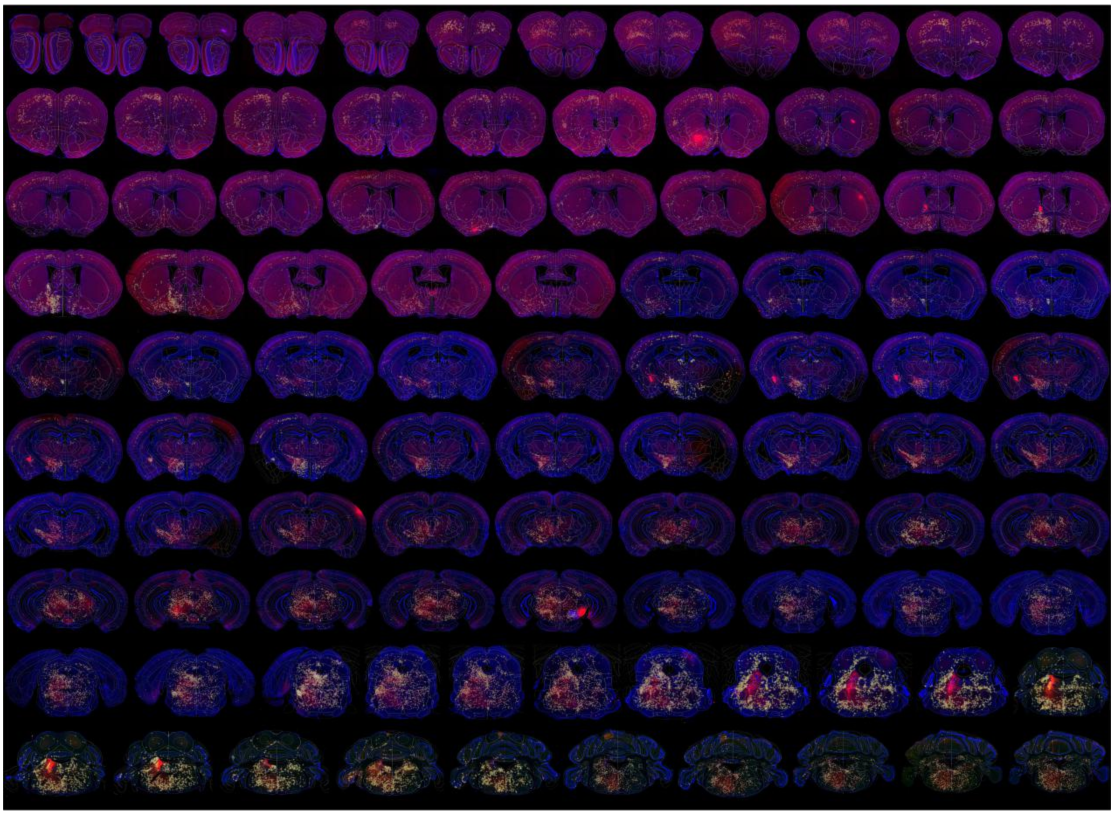

Supplementary Figure S4. Brain-wide image registration.

A representative male mouse brain (M1) with a total of 98 coronal sections ranging from bregma +3.0 to -6.36 mm were displayed sequentially to show the brain-wide distribution of presynaptic inputs to LC-NE neurons after the registration to the standard Allen Mouse Brain Reference Atlas.

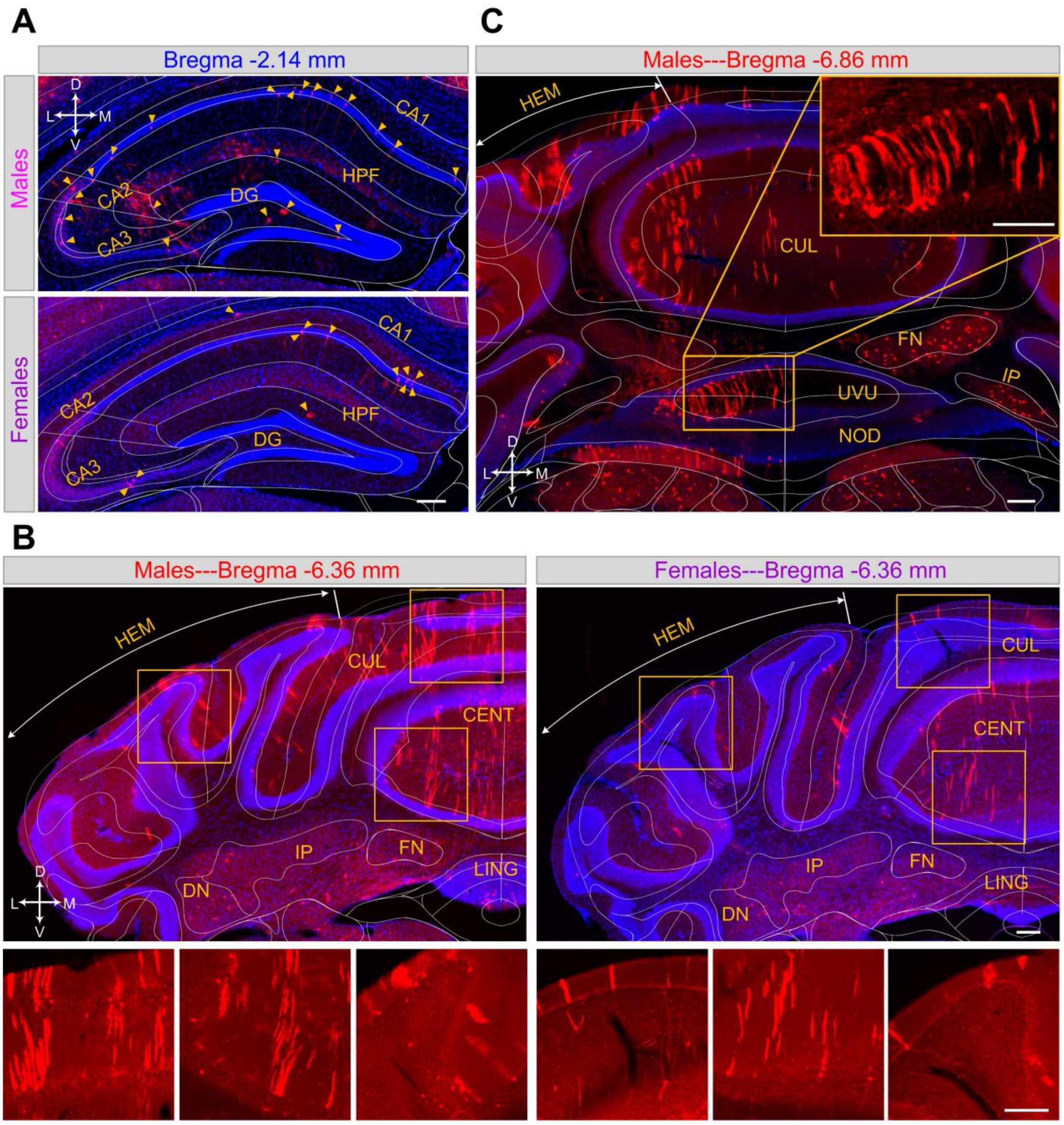

Supplementary Figure S5. Comparisons of inputs to male and female LC-NE neurons in hippocampus and cerebellar cortex.

(A) Representative images showing comparisons of inputs in dorsal HPF. Yellow arrowheads indicated the somas. Scale bars, 100 μm.

(B) Representative images showing comparisons of inputs in cerebellar cortex. Scale bars, 100 μm.

(C) Representative image showing input cells in cerebellar regions (male mice) beyond our presented data. Scale bars, 100 μm.

Abbreviations: CA1, field CA1; CA2, field CA2; CA3, field CA3; CENT, central lobule; CUL, culmen; DG, dentate gyrus; FN, fastigial nucleus; HEM, hemispheric regions; HPF, hippocampal formation; IP, interposed nucleus; LING, lingula; NOD, nodulus; UVU, uvula.
